# Evolutionary Analysis of MIKC^c^-Type MADS-Box Genes in Gymnosperms and Angiosperms

**DOI:** 10.3389/fpls.2017.00895

**Published:** 2017-05-30

**Authors:** Fei Chen, Xingtan Zhang, Xing Liu, Liangsheng Zhang

**Affiliations:** Center for Genomics and Biotechnology, Key Laboratory of Ministry of Education for Genetics, Breeding and Multiple Utilization of Crops, Fujian Provincial Key Laboratory of Haixia Applied Plant Systems Biology, Fujian Agriculture and Forestry UniversityFuzhou, China

**Keywords:** MADS-box genes, ABCE model, gymnosperms, basal angiosperms, molecular evolution, flowering

## Abstract

MIKC^c^-type MADS-box genes encode transcription factors that control floral organ morphogenesis and flowering time in flowering plants. Here, in order to determine when the subfamilies of MIKC^c^ originated and their early evolutionary trajectory, we sampled and analyzed the genomes and large-scale transcriptomes representing all the orders of gymnosperms and basal angiosperms. Through phylogenetic inference, the MIKC^c^-type MADS-box genes were subdivided into 14 monophyletic clades. Among them, the gymnosperm orthologs of *AGL6, SEP*, *AP1*, *GMADS*, *SOC1*, *AGL32*, *AP3*/*PI*, *SVP*, *AGL15*, *ANR1*, and *AG* were identified. We identified and characterized the origin of a novel subfamily *GMADS* within gymnosperms but lost orthologs in monocots and Brassicaceae. ABCE model prototype genes were relatively conserved in terms of gene number in gymnosperms, but expanded in angiosperms, whereas *SVP*, *SOC1*, and *GMADS* had dramatic expansions in gymnosperms but conserved in angiosperms. Our results provided the most detailed evolutionary history of all MIKC^c^ gene clades in gymnosperms and angiosperms. We proposed that although the near complete set of MIKC^c^ genes had evolved in gymnosperms, the duplication and expressional transition of ABCE model MIKC^c^ genes in the ancestor of angiosperms triggered the first flower.

## Introduction

MADS-box genes encode a family of transcription factors (TFs) that have fundamental roles in controlling the development in plants, animals, and fungi (Becker et al., 2000). Phylogenetic analysis of eukaryotic MADS-box genes identified the following two super clades: type I and type II genes. Type I TFs only contain one MADS domain, whereas type II TFs harbor an additional K-box at the C-terminal. MIKC^c^ and MIKC^∗^ together constitute the type II MADS-box genes in plants (Becker and Theißen, 2003). The split of MIKC^c^ and MIKC^∗^ genes happened in the ancestor of all land plants (Gramzow and Theissen, 2010). MIKC^c^ genes are concisely studied in expression patterns or mutant phenotypes and best known for their functions (Becker and Theißen, 2003), especially in flowering plants that work as floral organ identity genes. These floral organ identity MIKC^c^-type MADS-box genes have been subdivided into the following four classes: A, B, C, and E genes that provide five different homeotic functions, with A specifying sepals, A+B+E for petals, B+C+E for stamens, C+E for carpels, and D (sister of C genes) for ovules (Angenent and Colombo, 1991; Weigel and Meyerowitz, 1994).

A phylogenomic study of 17 plants, including eudicots, monocots, spike moss (*Selaginella moellendorffii*), and *Physcomitrella patens* identified 1,295 MADS-box genes, and classified MIKC^c^ genes into the following 14 clades: *StMADS11*, *AGL17*, *AGL12*, *TM3*, *FLC*, *AGL6*, *AGL2*, *SQUA*, *AG*, *TM8*, *OsMADS32*, *DEF*/*GLO*, *GGM13*, *AGL15* (Gramzow and Theißen, 2013). An early study covering gymnosperms, *Gnetum* sp., and *Cycas* sp., revealed that *AG*, *AGL6*, *AGL12*, *DEF*+*GLO*, *GGM13*, *STMADS11*, and *TM3-like* genes very likely existed in the ancestor of angiosperms and gymnosperms (Becker and Theißen, 2003). A comprehensive analysis of three conifer genomes suggests 11–14 type II MADS genes in the most recent common ancestor of seed plants (Gramzow et al., 2014). Another study covering 27 flowering plants traced back to 11 seed plant specific MIKC^c^ clades (Gramzow and Theißen, 2015). However, the confidence and resolution on the early evolution of MIKC^c^ genes will be largely restricted and lead to conflict results without a comprehensive analysis of all orders of gymnosperms and basal angiosperms.

The gymnosperms, including conifers, cycads, ginkgo, and gnetophytes, belong to the seed bearing plants that do not produce flowers. Gymnosperm seeds are borne on the scales of cones such as with pine and spruce trees, rather than angiosperm seeds, which are encased in a fruit. Decoding the molecular genetics and evolution of reproductive organ formation is essential, however, gymnosperms usually have very large genomes, thereby hindered the deciphering gymnosperm genomes. Up to date (November 30, 2016), only the genomes of *Picea abies* (Nystedt et al., 2013) and *Ginkgo biloba* (Guan et al., 2016) have been reported in gymnosperms. In *P. abies*, among 278 putative MADS-box genes, only 41 genes were expressed (Nystedt et al., 2013).

Flowering plants are involved in our daily lives including energy, material, food, and culture. In taxonomy, which plant, the amborella or water lily is the most basal angiosperm remains as a great abominable mystery. Thereby the sampling without a water lily genome would probably lead to wrong result when studying the MIKC^c^ genes in these basal angiosperms and even in implicating the number of clades in the ancestor of seed plants. Actually, basal angiosperms constitute about 175 species from the following three orders: Amborellales, Nymphaeales, and Austrobaileyales (Zeng et al., 2014), in which only the genome sequence of *Amborella trichopoda* was released. Thirty-six *Amborella* MIKC^c^ MADS-box genes were identified and classified into main clades such as *AP1*, *AGL6*, *AGL2*, *AGL9*, *AP3*, *PI*, *AG*, *STK* (Project, 2013), underscoring the importance of *Amborella* for understanding the evolution of MADS-box genes.

In this study, we relied on the recently released genomes and large-scale of transcriptomes of both gymnosperms and angiosperms (sampling from all orders of gymnosperms and basal angiosperms) to characterize and analyze the MIKC^c^ MADS-box genes. We identified the complete set of MIKC^c^ MADS-box genes from gymnosperms and basal angiosperms. The expression of ginkgo MIKC^c^ MADS-box genes in reproductive organs was also studied. We report the MIKC^c^ MADS-box genes from samples of each order of seed plants, laying the foundation for functional analysis of the early evolution of flower formation and gymnosperm reproductive organ formation.

## Materials and Methods

### Data Retrieval

The genome and transcriptome data of *G. biloba* were downloaded from the ginkgo genome sequencing project (gigadb.org/dataset/100209). The available genome of *Pinus taeda* (Neale et al., 2014) was downloaded from congenie.org. *Pinus sylvestris*’s genome was downloaded from dendrome.ucdavis.edu/treegenes. We also performed blast search against the 6,337 proteins from Cycadales species and 1,924 proteins from Gnetales species from NCBI’s protein database^1^. The water lily *Nymphaea colorata* genome was recently sequenced by our own sequenced genome project and could be found from our database www.angiosperms.org (unpublished). The genome of *Amborella* was downloaded from www.amborella.org. All the other MADS-box genes from transcriptome sequences were downloaded from OneKP project (Matasci et al., 2014). All four orders of gymnosperms (Pinales, Ginkgoales, Cycadales, Gnetales) and all three orders of basal angiosperms Nymphaeales, Amborellales, Austrobaileyales were covered in this study. The sampled species with abbreviations were listed in **Table 1**.

**Table 1:** Samples used in this study and the number of MIKC^c^ genes found in each plant.

Clade	Order	Family	Species	Gene symbol	MIKC^c^
	Amborellales	Amborellaceae	*Amborella trichopoda*	URDJ	7
	Amborellales	Amborellaceae	*Amborella trichopoda^∗^*	scaffold	15
	Nymphaeales	Nymphaeaceae	*Nymphaea colorata^∗^*	Nym	13
	Nymphaeales	Nymphaeaceae	*Nymphaea* sp.	PZRT	3
	Nymphaeales	Nymphaeaceae	*Nuphar advena*	WTKZ	2
	Austrobaileyales	Austrobaileyaceae	*Austrobaileya scandens*	FZJL	10
	Austrobaileyales	Schisanclraceae	*Kadsura heterodita*	NWMY	3
Angiosperms	Austrobaileyales	Schisanclraceae	*Illicium parviflorum*	ROAP	5
	Austrobaileyales	Schisanclraceae	*Illicium floridanum*	VZCI	9
	Ceratophyllales	Ceratophyllaceae	*Ceratophyllum demersum*	NPND	4
	Vitales	Vitaceae	*Vitis vinifera^∗^*	VIT	31
	Brassicales	Brassicaceae	*Arabidopsis thaliana^∗^*	At	37
	Malpighiales	Salicaceae	*Populus trichocarpa^∗^*	Potri	56
	Poales	Bromeliaceae	*Ananas comosus^∗^*	Aco	25
	Poales	Poaceae	*Oryza sativa^∗^*	Loc OS	32
	Poales	Poaceae	*Sorghum bicolor^∗^*	Sorbic	30
	Cycadales	Zamiaceae	*Encephalartos barteri*	GNQG	3
	Cycadales	Stangeriaceae	*Stangeria eriopus*	KAWQ	4
	Cycadales	Zamiaceae	*Dioon edule*	WLIC	2
	Cycadales	Cycadaceae	*Cycas micholitzii*	XZUY	4
	Ginkgoales	Ginkgoaceae	*Ginkgo biloba*	SGTW	10
	Gnetales	Gnetaceae	*Gnetum montanum*	GTHK	6
	Gnetales	Welwitschiaceae	*Welwitschia mirabilis*	TOXE	3
	Gnetales	Ephedraceae	*Ephedra sinica*	VDAO	2
	Pinales	Pinaceae	*Pines taeda^∗^*	PITA	12
	Pinales	Pinaceae	*Pines sylvestris^∗^*	MA	5
	Ginkgoales	Ginkgoaceae	*Ginkgo biloba^∗^*	Gb	11
	Pinales	Podocarpaceae	*Falcatifolinm taxoides*	ROWR	11
	Pinales	Cupressaceae	*Chamaecyparis lawsoniana*	AIGO	11
	Pinales	Pinaceae	*Pseudolarix amabilis*	AQFM	11
	Pinales	Pinaceae	*Nothotsuga longibracteata*	AREG	7
	Pinales	Cupressaceae	*Widdringtonia cedarbergensis* AUDE		8
	Pinales	Pinaceae	*Picea engelmannii*	AWQB	9
	Pinales	Podocarpaceae	*Microstrobos fitzgeraldii*	BBDD	2
	Pinales	Taxaceae	*Austrotaxus spicata*	BTTS	6
	Pinales	Cupressaceae	*Platycladus orientalis*	BUWV	9
	Pinales	Podocarpaceae	*Manoao colensoi*	CDFR	7
	Pinales	Cupressaceae	*Tetraclinis* sp.	CGDN	6
	Pinales	Pinaceae	*Pinus radiata*	DZQM	9
	Pinales	Taxaceae	*Torreya taxifolia*	EFMS	10
	Pinales	Podocarpaceae	*Prumnopitys andina*	EGLZ	10
	Pinales	Cupressaceae	*Pilgerodendron uviferum*	ETCJ	10
	Pinales	Cupressaceae	*Taxodium distichum*	FHST	12
	Pinales	Podocarpaceae	*Dacrycarpus compactus*	FMWZ	8
	Pinales	Cupressaceae	*Calocedrus decurrens*	FRPM	10
	Pinales	Pinaceae	*Tsuga heterophylla*	GAMH	8
	Pinales	Pinaceae	*Cedrus libani*	GGEA	11
	Pinales	Cupressaceae	*Diselma archeri*	GKCZ	10
	Pinales	Taxaceae	*Torreya nucifera*	HQOM	11
	Pinales	Cephalotaxaceae	*Amentotaxns argotaenia*	IAJW	4
	Pinales	Cupressaceae	*Callitris gracilis*	IFLI	4
	Pinales	Pinaceae	*Pinus parviflora*	IIOL	19
Gymnosperms	Pinales	Pinaceae	*Pseudotsuga wilsoniana*	IOVS	11
	Pinales	Podocarpaceae	*Dacrydium balansae*	IZGN	10
	Pinales	Pinaceae	*Pinus ponderosa*	JBND	9
	Pinales	Cupressaceae	*Neocallitropsis pancheri*	JDQB	7
	Pinales	Podocarpaceae	*Phyllocladus hypophyllus*	JRNA	10
	Pinales	Pinaceae	*Keteleeria evelyniana*	JUWL	12
	Pinales	Podocarpaceae	*Parasitaxus usla*	JZVE	9
	Pinales	Podocarpaceae	*Sundacarpus amarus*	KLGF	8
	Pinales	Pinaceae	*Pines jeffreyi*	MFTM	9
	Pinales	Podocarpaceae	*Microcachrys tetragona*	MHGD	8
	Pinales	Araucariaceae	*Agathis robusla*	MIXZ	4
	Pinales	Pinaceae	*Cathaya argyrophylla*	NPRL	13
	Pinales	Cupressaceae	*Metasequoia glyptostroboides* NRXL		16
	Pinales	Cupressaceae	*Cunninghamia lanceolata*	OUOI	11
	Pinales	Cupressaceae	*Papuacedrus papuana*	OVIJ	9
	Pinales	Podocarpaceae	*Halocarpus bidwillii*	OWFC	12
	Pinales	Podocarpaceae	*Falcatifolium taxoides*	PLYX	5
	Pinales	Podocarpaceae	*Saxegothaea conspicua*	QCGM	6
	Pinales	Cupressaceae	*Sequoiadendron giganteum*	QFAE	4
	Pinales	Podocarpaceae	*Falcatifolium taxoides*	QHBI	3
	Pinales	Cupressaceae	*Cupressus dupreziana*	QNGJ	8
	Pinales	Cupressaceae	*Taiwania cryptomerioides*	QSNJ	9
	Pinales	Cupressaceae	*Callitris macleayana*	RMMV	6
	Pinales	Araucariaceae	*Wollemia nobilis*	RSCE	8
	Pinales	Podocarpaceae	*Podocarpus coriaceus*	SCEB	8
	Pinales	Cupressaceae	*Fokienia hodginsii*	UEVI	10
	Pinales	Podocarpaceae	*Nageia nagi*	UUJS	5
	Pinales	Podocarpaceae	*Retrophyllum minus*	VGSX	10
	Pinales	Pinaceae	*Abies lasiocarpa*	VSRH	8
	Pinales	Pinaceae	*Larix speciosa*	WVWN	9
	Pinales	Taxaceae	*Taxus baccata*	WWSS	4
	Pinales	Cupressaceae	*Athrotaxis cupressoides*	XIRK	8
	Pinales	Podocarpaceae	*Podocarpus rubens*	XLGK	7
	Pinales	Cupressaceae	*Juniperus scopulorum*	XMGP	5
	Pinales	Cupressaceae	*Microbiota decussata*	XQSG	15
	Pinales	Sciaclopityaceae	*Sciadopitys verticillata*	YFZK	4
	Pinales	Taxaceae	*Pseudotaxus chienii*	YLPM	10
	Pinales	Cupressaceae	*Austrocedrus chilensis*	YYPE	13

*^∗^Indicates this species has the available genome sequence.*

### Identification of MADS-Box Genes

For those genome sequences, MADS-box genes were predicted using HMMER software (Finn et al., 2011) with the seeds built based on an alignment of reliable MADS genes from all groups of MADS-domain proteins from the representative species *Arabidopsis thaliana*, *Oryza sativa*, and *P. abies*. For the transcriptome sequences in OneKP database^2^, redundant sequences were already removed. MADS-box genes were predicted using BLASTP tool (Altschul et al., 1990) using the functional annotated *Arabidopsis* orthologs as the seeds against the OneKP database.

### Sequence Alignment and Phylogenetic Analysis

Multiple sequences were aligned using the accurate alignment software MAFFT (Katoh and Standley, 2013) with default parameters. For the large alignment, the fast and accurate near maximum-likelihood phylogenetic trees were constructed using FastTree software using the JTT+CAT model (Price et al., 2009). In the phylogenetic tree, supporting values below 50 were generally regarded unreliable and hided.

## Results

### Major Clades of MIKC^c^ MADS-Box Genes in Gymnosperms

**FIGURE 1:**
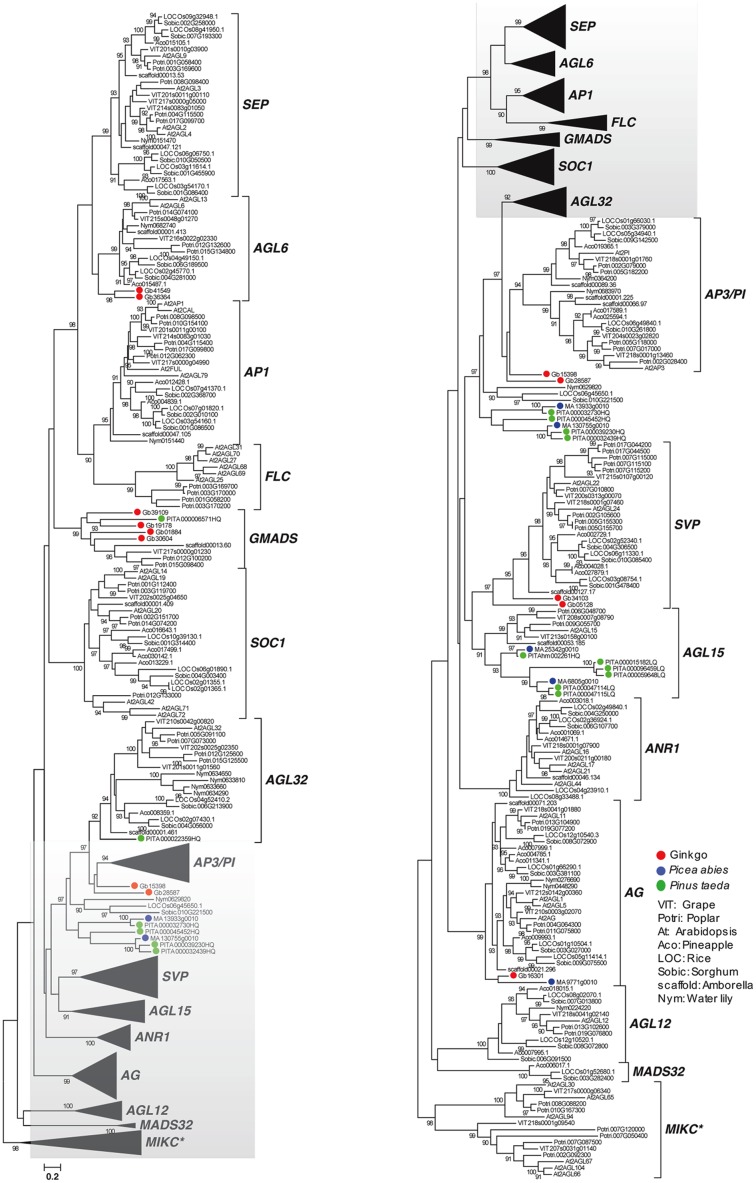
Genome-wide analysis of MIKC^c^ MADS-box genes using gymnosperm, basal angiosperm, and crown angiosperm genomes. Sequences from gymnosperms were labeled = with the following colors: red for ginkgo, green for *Pinus taeda*, and blue for *Picea abies*.

Based on the survey of three gymnosperm (*P. taeda, P. sylvestris, G. biloba*) and eight angiosperm (*Vitis vinifera, A. thaliana, Populus trichocarpa, Ananas comosus, O. sativa, Sorghum bicolor, A. trichopoda, N. colorata*) genomes, the following 14 major clades of MIKC^c^ MADS-box genes were characterized: *SEP*, *AGL6*, *AP1*, *FLC*, *GMADS*, *SOC1*, *AGL32*, *AP3*/*PI*, *SVP*, *AGL15*, *ANR1*, *AG*, *AGL12*, *MADS32* (**Figure 1**). However, gymnosperm genes were distributed into the following six clades: *AGL6*, *GMADS*, *AGL32*, *SVP*, *AGL15*, *AG*. This is partly because only 12, 5, 11 MIKC^c^ MADS-box genes found in *P. taeda*, *P*. *sylvestris*, *G. biloba*, respectively (**Table 1**). These 28 MIKC^c^ MADS-box gymnosperm genes might be useful in studying the early evolution of MIKC^c^ MADS-box genes, however, their limited number may not have a full coverage, and may lead to incomplete evolutionary reconstruction.

### Refinement of Clade Classification Using Large-Scale Transcriptome Data

Due to the limited genomic sequences of gymnosperms and basal angiosperms, the transcriptome data of often-neglected species covering gymnosperms and basal angiosperms was employed (**Table 1**) to reveal the evolutionary details. These include 8 basal angiosperms covering all three orders and 71 gymnosperms from all the orders of gymnosperms. We identified 623 MIKC^c^ MADS-box genes from the basal angiosperm and gymnosperm transcriptomes. In the gymnosperm transcriptomes alone 580 MIKC^c^ MADS-box genes were identified, which was 20-fold more than those from three gymnosperm genomes.

**FIGURE 2:**
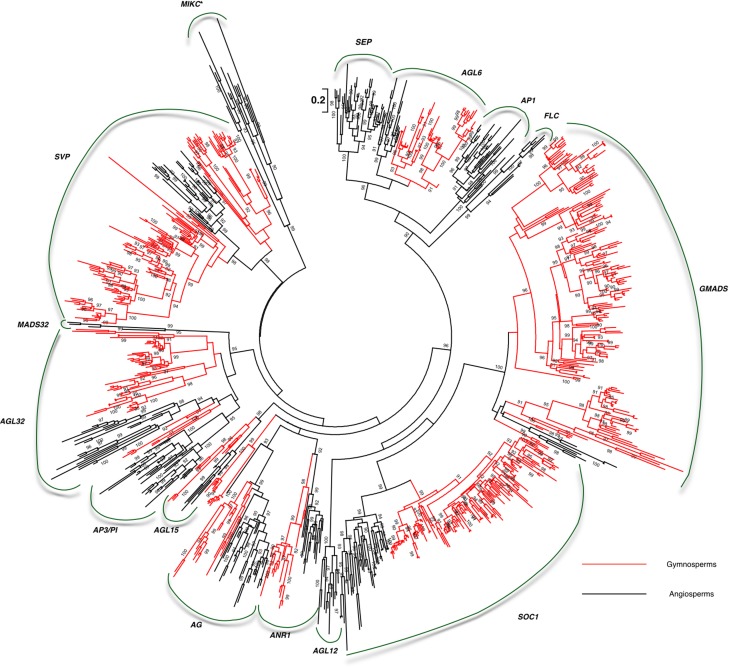
Large-scale transcriptomes revealed the early evolution of MIKC^c^ MADS-box genes in gymnosperms and basal angiosperms. Branches colored in red indicate gymnosperm sequences.

Relying on more gymnosperm and angiosperm sequences, the details of the characterized clades were revealed. The 13rd clade MADS32 with sequences from both basal angiosperm *Amborella* and three monocots was also detected. No *Arabidopsis* genes were found in the monophyly (**Figure 2**). Due to the limited information, we proposed to name this clade of genes as *MADS32* based on the name of a rice ortholog *OsMADS32*. So taken together, the following 14 MIKC^c^ clades were characterized from basal angiosperms and gymnosperms: *SVP*, *MADS32*, *AP3*/*PI*, *AGL32*, *AGL15*, *AG*, *ANR1*, *AGL12*, *SOC1*, *GMADS*, *FLC*, *AP1*/*FUL*, *AGL6*, and *SEP* (**Figure 2**).

Since different researchers preferred their own nomenclature standards in classifying the MIKC^c^ MADS-box genes, which often leads to confusion for the public or beginners. We listed the current representative classifications in **Table 2**. Currently, we have identified all the reported clades and refined several clades based on more representative sequences from all orders of gymnosperms and angiosperms.

**Table 2:** Classifications of MIKC^c^ from several reports.

Shan et al., 2009; Xue et al., 2010	Gramzow et al., 2014	Heijmans et al., 2012	Our report
*AP1*			*AP1*
*SEP*		*SQUA, SEP*	*SEP*
*AGL6*		*AGL6*	*AGL6*
*FLC*	*AGL2/AGL6/SQUA/FLC*	*FLC*	*FLC*
*AGL12*	*AGL12*	*AGL12*	*AGL12*
*AG*	*AGAMOUS*	*AG, FBP11*	*AG*
*S0C1*	*TM3*	*TM3*	*SOC1*
*SVP*	*StMADS11*	*STMADS11*	*SVP*
*ANR1*	*AGL17*	*AGL17*	*ANR1*
*AGL15*	*AGL15, GpMADS4*	*AGL15*	*AGL15*
*AP3/PI*			*AP3/PI*
*AGL32*	*DEF/GLO/OsMADS32/GGI∖GGM13, GLO, DEF, TM6*		*AGL32, MADS32*
	*TM8*	*TM8*	*GMADS*
Sum = 12 clades	Sum = 10 clades	Sum = 16 clades	Sum = 14 clades

*The representative reports (Shan et al., 2009; Xue et al., 2010; Heijmans et al., 2012; Gramzow et al., 2014) on the classifications and our proposed classification were also shown.*

### Evolution of A-, B-, C-, E-Function Genes

**FIGURE 3:**
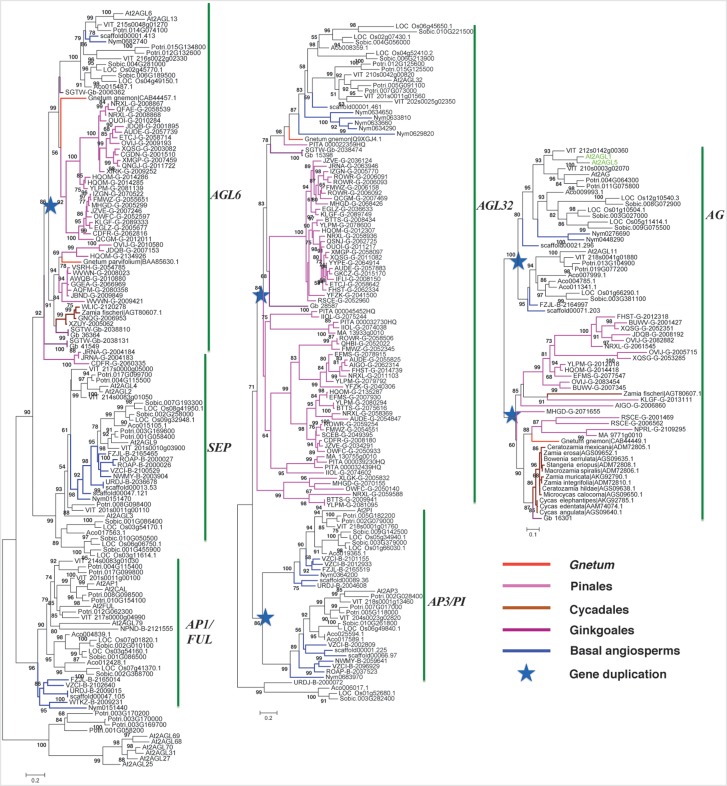
Phylogeny of the ABCE model genes in gymnosperms and angiosperms. Branches colored in blue, purple, red, magenta, and coffee-color indicate basal angiosperm, ginkgo, Gnetales, Pinales, and Cycadales sequences, respectively. *Arabidopsis* sequences were colored in green. The star indicates the gene duplication event.

A-function *AP1*/*FUL* genes were only found in angiosperms. In the basal angiosperm stage, *AP1*/*FUL* genes retained a single copy in both sequenced *Amborella* and *Nymphaea* genomes showing the conserved evolution trajectory (**Figure 3**). However in monocots, two groups were found in the near-basal monocot pineapple (*Ananas comosus*) and they diverged into three groups in the crown monocots rice and sorghum. In the eudicots, three groups were clearly identified from basal plant grape to crown plant *Arabidopsis*, leading to the origin of divergence of *AP1* and *FUL* genes.

*SEP* and *AGL6* formed a very close sister group (**Figure 3**). *SEP* clade consisted only angiosperm genes, whereas *AGL6* was made-up of one group of angiosperm genes, and two groups of gymnosperm genes and suggests *SEP* and *AGL6* diverged within the ancestor of seed plants. Moreover, because basal gymnosperm (ginkgo) genes were found in both groups of *AGL6*, which suggests a duplication event occurred in the ancestor of gymnosperm and contributed the two groups. In the *AGL6* clade, only one group of angiosperm genes was found, whereas three groups of angiosperm genes were found in the *SEP* clade because of the sampling from basal angiosperms to crown angiosperms. Two groups of *SEP* genes contained genes from basal angiosperms, monocots and eudicots, but the third group of *SEP* only contained monocot genes.

*AP3* encodes a MADS-box protein that specifies petal and stamen identities, and *PI* encodes a MADS-box required for the specification of petal and stamen identities. The two groups, *AP3* and *PI*, each consisted of genes from both basal and crown angiosperms (**Figure 3**), suggesting that they diverged in the ancestor of angiosperms, which were most likely yielded by the angiosperm specific whole genome duplication (WGD).

*AGL32* consisted of genes from both gymnosperms and angiosperms (**Figure 3**), suggesting it originated in the ancestor of seed plants. In gymnosperms, it radiated into three groups, which was not revealed analyzing the three genomes. In angiosperms, *AGL32* had two groups in the rosids.

C-function genes were the sister groups of *AG* in the angiosperms, but they had close orthologs in the gymnosperms (**Figure 3**). Although this C clade in basal gymnosperm preserved a single copy of the genes, they were duplicated in crown gymnosperms. For example, in species classified in the Pinales three copies were found in *Papuacedrus papuana* (with gene identity: OVIJ), two copies were identified in *Microbiota decussata* (XQSG) and *Platycladus orientalis* (BUWV).

### Expansion of *SVP*, *SOC1*, and *GMADS* Genes in Gymnosperms

*SVP*, *SOC1*, and *GMADS* are generally not regarded as core genes involved in the floral organ formation, and only very limited information of these clades are available. However, the genes are essential for other aspects for flowering, such as the agents for flowering time control [*SVP* (Li et al., 2015); *SOC1* (Liu et al., 2016); (Gao et al., 2016)]. Therefore, we set-out to characterize their evolutionary trajectory.

### SVP

*SVP* encodes a MADS-box TF acting as a central regulator of flowering time, and were found in both gymnosperms and angiosperms (**Figure 2**). In angiosperms, only one monophyletic group was evolved. Full coverage of data showed that *SVP* retained a single copy in basal angiosperms, however, they duplicated in tree groups of the Poaceae and three groups in rosid. In gymnosperms, two monophylic groups of *SVP*s were clustered. *SVP*s were subdivided into two groups in group I, and further divided into seven groups in group II. *SVP* expanded into more copies in gymnosperms than that in angiosperms. SVP genes were expressed in young shoot, young shoot, leaf, young leaf both in basal angiosperms and gymnosperms as detected in transcriptome sequencing.

### SOC1

Although *SOC1* was not found in the three genomes of gymnosperms (**Figure 1**), 141 gymnosperm *SOC1* genes were recognized. Phylogenetic analysis showed that 10 groups were identified in the crown Pinales species. Unlike the dramatic expansion of *SOC1* in gymnosperms, basal-most angiosperms, Amborella, had only a single copy (**Figure 2**). However, basal angiosperms *Austrobaileya scandens* (FZJL) and *Illicium floridanum* (VZCI), *Illicium parviflorum* (ROAP) all had duplicated into two subgroups, which nested together, and had a complicated evolutionary history. In crown angiosperms, three subgroups of *SOC1* were found in eudicots and two subgroups identified in monocots.

### GMADS

We found a highly supported monopyly with supporting values 99. In this monophyly, no *Arabidopsis* ortholog was detected, which possibly led to its neglect of characterization (**Figure 1**). This monophyly covers orthologs from gymnosperms, basal angiosperms, and eudicots, showing the consistency of its evolutionary history. Transcriptome data was then employed, which covered gymnosperms and basal angiosperms (**Table 1**), to reveal the evolutionary details. MIKC^c^ genes were found among the transcriptomes of 8 basal angiosperms and 71 gymnosperms (**Table 1**). We identified 188 members from the novel subfamily, making it the largest subfamily among all MADS-box gene family (**Figure 2**). Besides, 10 subgroups were characterized in the crown gymnosperms. Because determining the phylogenetic relationship of this novel clade with other MADS-box genes was difficult and its lack of name, we proposed to name this clade of genes as *GMADS* for its significant expansion in gymnosperms.

### Evolution Atlas of All MIKC^c^ Clades in Seed Plants

**FIGURE 4:**
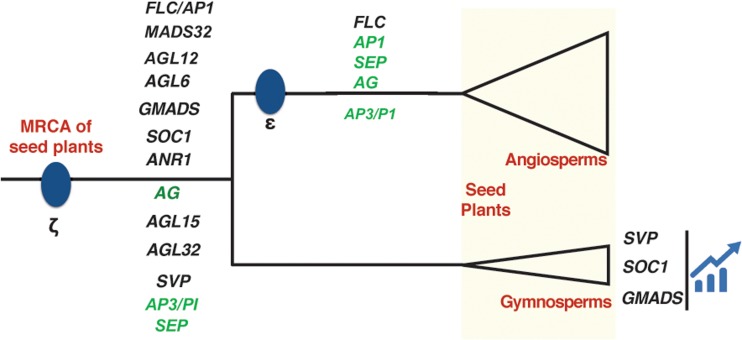
Evolution of MIKC^c^ clades plotted on a phylogenetic tree of seed plants. Two whole genome duplication (WGD) events ζ and 𝜀 have been marked in blue dots. Three clades expanded significantly in gymnosperms were also presented. The ABCE genes were colored in green.

Among all the 14 clades, we found 8 clades *AGL6*, *GMADS*, *SOC1*, *ANR1*, *AG*, *AGL15*, *AGL32*, *SVP* cover multiple sequences from both gymnosperms and angiosperms (**Figure 4**), suggesting they originated at least in the most recent common ancestor of seed plants. The following seven clades SEP, FLC, AGL32, AP1, AGL12, AP3/PI, MADS32 were identified only in angiosperms. No clade was specific to gymnosperms (**Figure 4**).

### Expressional Profiling of Ginkgo MIKC^c^-Type MADS-Box Genes

The gingko genome encoded 11 MIKC^c^-type MADS-box genes, which was distributed into the following five clades: *AGL6*, *GMADS*, *AP3*/*PI*, *SVP*, *AG*, and covered three major functional clades A/E, B, C. Because the genome has been recently sequenced (Guan et al., 2016), *G. biloba* serves as a good model for functional and comparative studies. The expression of these 11 genes was quantified using three transcriptomes covering reproductive (male and female) and vegetative (stem and leaf) organs, and all the 11 genes were well-quantified in the transcriptomes. *AG* (*Gb_16301*), *AGL6* (*Gb_41549*), *AP3*/*PI* (*Gb_28587*), *GMADS* (*Gb_01884* and *Gb_30604*) genes were specifically expressed in the reproductive organs with no expression detected in the vegetative organs stem and leaf (**Table 3**). The four *GMADS* genes were expressed significantly higher in reproductive organs than in vegetative organs. The two ginkgo *SVP* orthologs had divergent expression patterns. *Gb_05128* was expressed strongly in reproductive organs, whereas *Gb_34103* had strong expression in both reproductive and vegetative organs, which suggested their divergent functions.

**Table 3:** Expressional profile of 11 ginkgo MIKC^c^ genes.

MADS-box clade	Gene Id	Female reproductive organ	Male reproductive organ	Leaves and stems of seedlings
*AG*	*Gb 16301*	80.83	290.18	0
*AGL6*	*Gb 41549*	23	37.79	0
*AGL6*	*Gb 36364*	28.77	115.6	0.92
*AP3/PI*	*Gb 28587*	0	0.18	0
*AP3/PI*	*Gb 15398*	32.13	4.25	16.83
*GMADS*	*Gb 01884*	25.53	87.12	0
*GMADS*	*Gb 39109*	1.29	5.02	4
*GMADS*	*Gb 19178*	1.62	1.31	0.94
*GMADS*	*Gb 30604*	15.34	79.43	0
*SVP*	*Gb 05128*	19.41	46.73	0.99
*SVP*	*Gb 34103*	0.63	49.01	51.48

## Discussion

### The Gymnosperm and Angiosperm MIKC^c^-Type MADS-Box Genes

Although the MIKC^c^ genes were detected in the crown gymnosperm *P. abies* and other three Pinales species (Gramzow et al., 2014), genes from a single gymnosperm order could not represent the ancestral state of gymnosperms made up of four orders. High resolution and systematic analysis of basal angiosperm and gymnosperm MIKC^c^ MADS-box genes is lacking due to the lack of omics data in previous studies. In this study, all the orders of gymnosperms and basal angiosperms were sampled, to show the high resolution of early evolution of MIKC^c^-type MADS-box genes. Our preliminary study using three gymnosperm genomes identified the presence of gymnosperm orthologs in clades *AGL6*, *GMADS*, *AGL32*, *AP3*/*PI*, *SVP*, *AGL15*, *AG*. In addition, large-scale transcriptome data revealed gymnosperm orthologs from *SEP*-*AGL6*-*AP1* group, *SOC1*, *ANR1*, *AGL12*. *OsMADS32* clade was reported to be monocot specific (Sang et al., 2012), however, our transcriptome analysis revealed an ortholog from *Amborella*, which was not found in *Amborella* genome, supporting the high resolution of our transcriptome sampling. The *GpMADS4-like* gene clade was thought to be gymnosperm specific (Gramzow et al., 2014), however, it is part of AGL15 in our classification with supporting value 98 in the tree, suggesting the accuracy of our phylogeny.

Gymnosperms often have very large genomes, however polyploidy, usually leading to rapid increase in genome size, is rare among in this group (Gramzow et al., 2014). Only 28 MIKC^c^ MADS-box genes were found in genome sequenced *P. taeda*, *P*. *sylvestris*, *G. biloba*, collectively. In contrast, 38 genes were found in *O. sativa* (Arora et al., 2007), 37 in *A. thaliana* (Becker and Theißen, 2003), and 38 in *Vitis vinifera* (Díaz-Riquelme et al., 2009), which are significantly standing out compared to those very large gymnosperm genomes. In basal angiosperms, 15 and 13 MIKC^c^ MADS-box genes were detected in *Amborella* and water lily *N. colorata*, respectively. All these lines of evidences suggest that WGD contribute greatly to its expansion in crown angiosperms.

Specifically, *SOC1, SVP, GMADS* clades expanded greatly in gymnosperms and no functional study has been reported in gymnosperms. *GMADS* might control specific and unknown roles in gymnosperm reproductive organ development based on their expressional analysis in this study. Limited expression in vegetative tissues such as leaf and stem of *GMADS* and *SVP* genes were also reported in this study and previous report (Gramzow et al., 2014). The *SOC1* genes (or *TM3-like*) were also reported to have expression in both vegetative and reproductive organs (Gramzow et al., 2014). Considering their vital roles in regulating flowering time in angiosperms, we propose that among their diverse roles that triggering the reproductive organ development by *GMADS* and *SVP* genes in gymnosperms be included. In summary, the near complete set of MIKC^c^ type MADS-box genes in gymnosperms suggests the genetic material was the progenitor of the first flower.

### The ABCE Model Prototype Genes in Gymnosperms

After analyzing the ABC model genes in *P. abies*, A/G/E, B, C/D gene ancestors were present (Project, 2013), although only C-function gene was confirmed in the MRCA of seed plants reported. In basal angiosperms, *Eschscholzia californica*, *SEP* may have the same functions like *AP1* of A-function genes (Zahn et al., 2010). The A-class and E-class genes had two groups of orthologs in gymnosperms. So, we hypothesized that gymnosperm *AGL6* orthologs may have functions in reproductive organ formation. Our hypothesis is supported by expressional analysis of a ginkgo *AGL6* ortholog *Gb_36364*, which had very high expression in both male and female reproductive organs. We also hypothesized that the two diverged gymnosperm *AGL6* groups will have different functions, similar to the functional divergence of A-function and E-function genes, which needs future functional analysis.

For *AP3*/*PI* genes controlling the B-functions, and *AG* genes controlling the C- and D-functions, gymnosperm ancestors were traced back to as early as the emergence of ginkgo. No orthologs were identified in the Cycadales, another gymnosperm early branch. These genes were specifically expressed in reproductive organs and not detected in the transcriptome. A high quality genome from Cycadales species will be highly favored. In angiosperms, the heterodimerization of AP3 and PI proteins is necessary for B-function (Project, 2013). We have detected the gene duplication of AGL32 orthologs in gymnosperms, and the duplicates only form homodimers in *Gnetum* and *Picea* (Project, 2013) suggesting that the protein-protein interaction form is a crucial step in the origin of B-function, but not gene duplication for angiosperms.

## Conclusion

In this report, we sampled and analyzed species from all the orders of gymnosperms and the less-visited basal angiosperms including both newly released genomes and high quality large-scale transcriptomes. The major MIKC^c^-type MADS-box genes were characterized and we identified a new clade *GMADS*. The ABCE model prototype genes were relatively conserved in terms of gene number in gymnosperms, but expanded in angiosperms. In contrast, *SVP*, *SOC1*, and *GMADS* have dramatic expansion in gymnosperms, but retained conserved in angiosperms. The expression atlas of all MIKC^c^ genes in various organs from ginkgo was measured for the first time in this study. Our results provided strong evidence for the early evolution of MIKC^c^ MADS-box genes and high resolution evolution trajectory, which will largely enhance our understanding of this key transcription family and shed light on decoding its functional correlation to reproductive organ formation in gymnosperms and angiosperms. This study also illustrated the near complete set of MIKC^c^ genes in gymnosperms and suggest that genome duplication, together with expressional transition of MIKC^c^ genes in the ancestor of flowering plants are the major contribution to the first flower.

## Author Contributions

LZ designed the research. FC and LZ collected and analyzed the data. FC, XZ, XL, and LZ wrote, revised, and approved the manuscript.

## Conflict of Interest Statement

The authors declare that the research was conducted in the absence of any commercial or financial relationships that could be construed as a potential conflict of interest.

## Footnotes

1 https://www.ncbi.nlm.nih.gov

2 http://db.cngb.org/blast4onekp/blast
